# Oxidative Stress in Cardiovascular Disease

**DOI:** 10.3390/ijms15046002

**Published:** 2014-04-09

**Authors:** Gábor Csányi, Francis J. Miller

**Affiliations:** 1 Vascular Medicine Institute, E1228-1B BST, University of Pittsburgh, 200 Lothrop Street, Pittsburgh, PA 15261, USA; 2 Departments of Internal Medicine and Anatomy and Cell Biology, University of Iowa, Veterans Affairs Medical Center, Iowa City, IA 52242, USA

**Keywords:** oxidative stress, reactive oxygen species, cardiovascular disease, redox signaling, oxidative biomarkers, antioxidant therapy

## Abstract

In the special issue “Oxidative Stress in Cardiovascular Disease” authors were invited to submit papers that investigate key questions in the field of cardiovascular free radical biology. The original research articles included in this issue provide important information regarding novel aspects of reactive oxygen species (ROS)-mediated signaling, which have important implications in physiological and pathophysiological cardiovascular processes. The issue also included a number of review articles that highlight areas of intense research in the fields of free radical biology and cardiovascular medicine.

Cardiovascular disorders (CVD) and their consequences account for one out of every three deaths in the United States, and CVD are the most serious health problem of the Western world [1]. With an ageing population and spread of the Western diet and lifestyle, the burden and medical costs of CVD will significantly increase worldwide in the coming decades. Although significant effort has been directed towards elucidating biomolecular events governing the initiation and progression of CVD, much remains unclear. As such, a better understanding of the mechanisms leading to CVD and their clinical consequences is urgently needed and one of the most serious challenges in medicine.

Reactive oxygen species (ROS) at physiological levels are now appreciated to function as signaling molecules to regulate a wide range of processes in the cardiovascular system and to contribute to the maintenance of cardiovascular homeostasis [2]. In contrast, excessive and/or sustained increase in ROS generation plays a pivotal role in the initiation, progression and clinical consequences of CVD [3–5]. Despite great progress in the field of free radical biology and advances in cardiovascular medicine, we still do not have a complete understanding of the underlying mechanisms of CVD and consequences of pathophysiologically elevated ROS in cardiovascular tissue. Much is still to be discovered regarding the mechanisms that control activation of individual ROS sources in vascular cells, the specific role of individual NADPH oxidase (Nox) isoforms in CVD, the crosstalk between Nox enzymes and other ROS sources, paracrine role of ROS and lipid peroxidation products, and identification of new redox targets and their pathophysiological role in CVD. Major efforts are also necessary to develop specific inhibitors of ROS sources suitable for clinical application.

This issue of the *International Journal of Molecular Sciences* focuses on the regulation of ROS sources in CVD, further explores how ROS interact with their downstream targets, identifies novel redox cell signaling pathways, and discusses emerging treatment strategies. The forum contains fourteen review papers and sixteen original research articles. The review papers were selected to highlight areas of intense research in the fields of free radical biology and cardiovascular medicine. For example, the review articles explore the possible links between systemic vascular disease and chronic obstructive pulmonary disorder [6], highlight potential new treatment strategies [7,8], and discuss the role of pathogens [9], dopamine receptors [10], receptor for advanced glycation endproducts (RAGE) [11], epidermal growth factor receptor [12], and protein glutathionylation [13] in CVD. Pitocco and colleagues discuss the role of oxidative stress in the pathogenesis of diabetes mellitus and its complications [14]. Additionally, Magenta *et al.* have provided a thorough overview of the mechanisms by which microRNAs regulate oxidative stress responses in CVD [15].

The first original research article in the special issue, by Feng *et al.*, evaluated the effects of α-lipoic acid treatment on the kidneys of non-obese Goto-Kakizaki rats that develop type 2 diabetes mellitus. The authors show that α-lipoic acid treatment decreased the mRNA expression of p22*^phox^* and p47*^phox^* in the kidney, increased glutathione levels, and attenuated the progression of diabetic nephropathy [16].

Obesity is becoming a global epidemic in both children and adults and it is associated with numerous CVD comorbidities such as systemic hypertension, stroke, heart disease, lipid abnormalities and atherosclerosis, and type 2 diabetes mellitus. A study by González-Muniesa *et al.* demonstrated that a given genetic background favoring a chronic disturbance of the metabolic homeostasis leads to upregulation of proinflammatory- and oxidative stress-related genes, which could underlie the development of obesity-associated diseases [17]. Dietary strategies and nutrient supplementation have been long used for the management of obesity and prevention of obesity-associated disorders. De la Iglesia *et al.* investigated the effectiveness of a new dietary strategy (energy restriction, a specific macronutrient distribution, high meal frequency, and high antioxidant capacity) in patients with obesity. The authors showed that this new diet attenuated levels of oxidative stress biomarkers, reduced android fat mass, and decreased blood pressure in obese patients [18]. Along the same lines, Tie *et al.* demonstrated that polypeptides isolated from achyranthes bidentata, a commonly used Chinese medicinal herb, reduce oxidative stress and exert cardioprotection following myocardial ischemia/reperfusion injury in rats [19]. Duarte and her co-workers showed that apigenin, an anti-inflammatory dietary flavonoid, inhibits lipopolysaccharide-induced endothelial cell apoptosis via restoring normal mitochondrial complex I activity, inhibiting mitochondrial ROS generation, and decreasing enzymatic activity of caspase-3 [20]. Interestingly, oleic acid supplementation has been shown to stimulate vascular endothelial growth factor (VEGF) synthesis and secretion in aortic vascular smooth muscle cells (VSMC) from lean Zucker rats via a mechanism involving increased ROS generation, and the actions of oleic acid were impaired in aortic VSMC from obese Zucker rats [21].

Recent studies demonstrated that a decrease in endogenous sulfur dioxide (SO_2_) production is associated with the development of CVD; however, the mechanisms responsible for this effect are not entirely clear [22]. In this issue, Jin *et al.* investigated the effects of an SO_2_ donor on myocardial injury and cardiac function in isopropylarterenol (ISO)-treated rats. The paper published by this group showed that SO_2_ treatment attenuates myocardial injury and improves cardiac function via inhibiting cardiomyocyte apoptosis [23].

Cardiovascular surgery exposes the heart and various blood vessels to prolonged periods of warm and cold ischemia. Wiedemann and his co-workers showed that analysis of mitochondrial function can be used as a suitable method for the assessment of cold ischemic injury [24]. Following electrical stimulation, cardiac myocytes isolated from senescence marker protein-30 knockout mice generated significantly more ROS compared to wild type controls, a mechanism that has been implicated in angiotensin II release and regulation of coronary vascular tone [25].

Advanced glycation end products (AGEs) play a pivotal role in the development and progression of diabetic heart failure. Brouwers *et al.* investigated whether reduction of AGEs by overexpression of the glycation precursor detoxifying enzyme glyoxalase-I (GLO-I) prevents diabetes-induced oxidative damage, inflammation, and fibrosis in the heart [26].

Al Ghouleh *et al.* utilized 2D Differential In-Gel Electrophoresis and Mass Spectrometry (2D-DIGE/MS) to identify new downstream targets of VSMC Nox1 signaling with significant translational potential [27]. Actin-related protein 2/3 complex subunit 2 (ARPC2), with no previous link to Nox isozymes, hydrogen peroxide, or other ROS, was identified downstream of Nox1-mediated p38 MAPK activation and demonstrated to play an important role in VSMC migration.

A research article by Cao *et al.* explored the mechanisms that regulate the metabolism of asymmetric dimethylarginine (ADMA), an endogenous inhibitor of nitric oxide synthase, in Dahl salt-sensitive rats [28]. They demonstrated that high salt intake decreases the expression and activity of dimethylarginine dimethylaminohydrolase (DDAH), an enzyme that metabolizes ADMA, leading to increased ADMA and lower plasma nitrite/nitrate levels.

Increasing evidence demonstrates that activated platelets are causally involved in the onset of vascular inflammatory processes and play a key role in the development of atherosclerosis [29]. In the present issue, Badrnya *et al.* showed that high-density lipoproteins (HDL) compete with the binding of oxidized low-density lipoprotein (oxLDL) to the platelet surface, leading to attenuated platelet activation and decreased ROS generation, mechanisms that may contribute to the atheroprotective and antithrombotic effects of HDL [30].

Deficiency of Nox subunits, such as p47*^phox^*, Nox1, or Nox2, in ApoE null mice attenuates lesion formation, demonstrating a critical role for Nox-derived ROS in the early stage of atherosclerosis [31–33]. Much less, however, is known about the role of Nox enzymes and ROS during plaque progression and the late complicated phase of the disease. The article, by Kinkade *et al.* demonstrated that apocynin treatment beginning at 17 weeks of age (*i.e*., after the development of atherosclerosis) and continued for an additional 17 weeks reduced vascular ROS levels and attenuated lesion progression in ApoE^−/−^/LDLR^−/−^ mice [34].

Finally, Hartono *et al.* showed that in a murine model of renal artery stenosis increased ROS generation occurs early in the disease, initiating a progressive cascade of events that lead to upregulation of oxidative stress-related genes, interstitial inflammation, renal fibrosis, and atrophy [35].

## Conclusions

Despite the enormous progress in the field of vascular free radical biology and cardiovascular medicine, many avenues remain to be explored. For example, we require a better understanding of the regulatory mechanisms responsible for oxidase activation in the heart and the vessel wall, the crosstalk between different sources of ROS, and their precise downstream targets and function. The development of new molecular tools, transgenic and knockout animals, and pharmacological agents that block the undesirable actions of pathophysiological ROS will also greatly advance the field of cardiovascular research. All manuscripts published in this special issue contributed to a better understanding of the regulation and function of ROS in CVD. The articles explored how ROS interact with their downstream targets, identified novel ROS targets, and discussed new strategies to attenuate oxidative stress in CVD. We remain committed to advancing knowledge of the free radical biology and cardiovascular fields and to identifying novel targets at which to intervene, so that more successful CVD therapies can be developed.
